# Clinical Characteristics and Antimicrobial Resistance Profiles of Pediatric Enterococcal Infections: A Comparison Between *Enterococcus faecium* and *Enterococcus faecalis*

**DOI:** 10.3390/pathogens15090927

**Published:** 2026-09-03

**Authors:** Zixuan Wang, Chenchen Li, Yuanjie Zhou, Xiang Deng, Weichun Huang, Qing Cao

**Affiliations:** 1 Department of Infectious Diseases, Shanghai Children’s Medical Center, Shanghai Jiao Tong University School of Medicine, Shanghai 200127, China; wangzixuan4258@163.com (Z.W.); zhouyuanjie@scmc.com.cn (Y.Z.); dx.sjy@sjtu.edu.cn (X.D.); 2 Department of Pediatric Surgery, Shanghai Children’s Medical Center, Shanghai Jiao Tong University School of Medicine, Shanghai 200127, China; lichenchen@scmc.com.cn; 3 Department of Laboratory Medicine, Shanghai Children’s Medical Center, Shanghai Jiao Tong University School of Medicine, Shanghai 200127, China

**Keywords:** *Enterococcus faecium*, *Enterococcus faecalis*, children, antimicrobial resistance, healthcare exposures

## Abstract

**Objective**: To compare the clinical characteristics, healthcare exposures, and antimicrobial susceptibility profiles of pediatric *Enterococcus faecium* and *Enterococcus faecalis* infections. **Methods**: This retrospective study included hospitalized children with *E. faecium* or *E. faecalis* isolated from sterile specimens between August 2018 and June 2025. Clinical characteristics and antimicrobial susceptibility were compared. Logistic regression identified factors associated with pathogen species, while negative binomial regression evaluated the association between species and length of hospital stay. **Results**: Among 167 patients, 98 had *E. faecium* infection and 69 had *E. faecalis* infection. No significant annual trend in species distribution was observed. Multi-site infection was more common with *E. faecium* than with *E. faecalis* (36.73% vs. 15.94%, *p* = 0.003). Hematologic malignancy, previous hospitalization, central venous catheterization, immunosuppressive therapy, higher procalcitonin levels, and longer hospitalization were more common in the *E. faecium* group. In three separate primary logistic regression models adjusted for septic shock, central venous catheterization and hospitalization for ≥14 days before culture positivity were associated with higher odds of *E. faecium* infection, whereas endotracheal intubation was associated with higher odds of *E. faecalis* infection. In post hoc sensitivity analyses jointly incorporating these healthcare exposures and either hematologic malignancy or immunosuppressive therapy, only central venous catheterization remained significantly associated with *E. faecium* infection. *E. faecium* infection was associated with a 33.8% longer hospital stay (aIRR = 1.338, 95% CI: 1.160–1.543, *p* < 0.001). *E. faecium* showed substantially greater resistance to ampicillin and penicillin. **Conclusions**: Pediatric *E. faecium* and *E. faecalis* infections differed in patient profiles, healthcare exposures, antimicrobial susceptibility, and disease burden. Species identification may support clinical assessment and antimicrobial stewardship.

## 1. Introduction

*Enterococcus* spp. are Gram-positive, facultatively anaerobic cocci that are constituents of the intestinal microbiota and are less commonly found in the vagina or oral cavity. They are important pathogens responsible for healthcare-associated and opportunistic infections. Clinically, enterococcal infections may manifest as bloodstream infections, intra-abdominal infections, urinary tract infections, wound infections, infective endocarditis, and other conditions. *Enterococci* exhibit strong environmental adaptability, can persist for prolonged periods in healthcare settings, and have a marked capacity for antimicrobial tolerance and for acquiring resistance. Therefore, they are of substantial clinical significance in patients with prolonged hospitalization, immunocompromising conditions, invasive procedures, or exposure to broad-spectrum antimicrobial therapy [1,2,3].

At the cellular level, enterococci are non-spore-forming, catalase-negative, ovoid Gram-positive bacteria that usually occur in pairs or short chains. Their thick peptidoglycan cell walls, cell-wall teichoic acids, and lipoteichoic acids contribute to cell integrity, surface adhesion, and host interactions, while their facultatively anaerobic metabolism and tolerance of bile salts, osmotic stress, and nutrient limitation facilitate intestinal colonization and environmental persistence. As commensals, enterococci participate in the intestinal microbial community; after antibiotic-associated dysbiosis or disruption of mucosal barriers, however, they can overgrow, translocate, and cause opportunistic infection [2,4,5].

In pediatric patients, enterococcal infections commonly occur in the context of severe underlying diseases, prolonged hospitalization, intensive care unit admission, surgical procedures, or immunosuppressive therapy. Previous studies have indicated that enterococci have evolved from being relatively uncommon pathogens to becoming important causes of healthcare-associated infections, a shift associated with an increasing population of susceptible hosts, more frequent invasive procedures, prolonged hospital stays, and widespread antimicrobial use [6]. Studies on pediatric enterococcal bloodstream infections have also suggested that these infections are predominantly healthcare-associated and frequently occur in children with severe underlying diseases [7,8,9,10].

*Enterococcus faecium* and *Enterococcus faecalis* are the two most common enterococcal species encountered in clinical practice, together accounting for more than 90% of clinical enterococcal isolates. From a taxonomic perspective, *E. faecalis* and *E. faecium* are closely related members of the genus *Enterococcus* but represent distinct species. Both are Gram-positive, catalase-negative, facultatively anaerobic cocci and share important biological characteristics, including gastrointestinal colonization, environmental persistence, biofilm formation, and the capacity to acquire antimicrobial-resistance determinants. They also produce similar results in several conventional laboratory tests, including bile-esculin hydrolysis, pyrrolidonyl arylamidase positivity, and growth in 6.5% sodium chloride. Consequently, Gram staining and a limited biochemical panel may establish an enterococcal phenotype without reliably differentiating the two species. Despite this overlap, their contrasting patterns of virulence, host association, and antimicrobial resistance may lead to differences in clinical context and therapeutic considerations. These shared and distinct features provide the clinical and biological rationale for directly comparing *E. faecalis* and *E. faecium* in pediatric invasive infections [2,4,11]. *E. faecalis* is usually the predominant enterococcal species colonizing the intestinal tract and is more commonly identified in general pediatric enterococcal infections, whereas *E. faecium* is more likely to exhibit antimicrobial resistance and transmission advantages in healthcare settings and immunocompromised hosts under antimicrobial selective pressure [9]. Previous studies have shown that *E. faecium* more commonly exhibits resistance to ampicillin and vancomycin than *E. faecalis*, whereas *E. faecalis* generally retains a high level of susceptibility to ampicillin [3,12].

*E. faecalis* and *E. faecium* differ in several biologically relevant traits. *E. faecalis* is generally more abundant in the healthy human intestine and may express virulence determinants such as aggregation substance, cytolysin, gelatinase, enterococcal surface protein, and pili that promote adhesion, biofilm formation, tissue invasion, or immune evasion. By contrast, hospital-adapted *E. faecium* lineages are characterized by marked genomic plasticity, acquisition of mobile genetic elements and antimicrobial-resistance determinants, altered penicillin-binding proteins (particularly PBP5), and surface adhesins that facilitate persistence in healthcare environments. These species-level differences provide a biological basis for the contrasting host profiles and antimicrobial susceptibility patterns examined in this study [2,4,5,13].

Numerous studies have investigated enterococcal infections and antimicrobial resistance in adults; however, studies focusing on differences in the clinical characteristics of pediatric infections caused by *E. faecium* and *E. faecalis* remain relatively limited. Clarifying differences in admitting department, underlying diseases, invasive procedures, infection severity, and antimicrobial susceptibility profiles may inform local risk assessment while culture and susceptibility results are pending. Therefore, using sterile-site cultures from hospitalized children at a single center, this study aimed to compare the clinical characteristics, associated healthcare exposures, and disease burden of *E. faecium* and *E. faecalis* infections. The study was designed to identify associations rather than causal relationships and to generate hypotheses for subsequent multicenter validation.

## 2. Materials and Methods

### 2.1. Study Population

This was a single-center retrospective study. Pediatric patients hospitalized at Shanghai Children’s Medical Center, from August 2018 to June 2025, in whom *E. faecium* or *E. faecalis* was isolated from sterile specimens, were included. Patients were divided into the *E. faecium* group and the *E. faecalis* group according to the species isolated.

The inclusion criteria were as follows:

① Age ≤ 18 years.

② Isolation of *E. faecium* or *E. faecalis* from sterile specimens during hospitalization.

③ Fulfillment of the clinical diagnostic criteria for infection. Infection was adjudicated based on clinical manifestations, laboratory findings, imaging examinations, and microbiological results, according to the Centers for Disease Control and Prevention/National Healthcare Safety Network (CDC/NHSN) surveillance definitions [14]. For suspected catheter-related bloodstream infection, the Infectious Diseases Society of America (IDSA) guideline for intravascular catheter-related infection was also consulted [15].

④ Complete clinical data.

The exclusion criteria were as follows:

① cases clinically judged as contamination or colonization;

② cases with missing key data;

③ cases with concomitant infection caused by other pathogens.

For patients with multiple positive cultures, only the first isolate meeting the diagnostic criteria for infection was included in the analysis.

### 2.2. Data Collection

Clinical data were collected through the electronic medical record system, including sex, age, admitting department, underlying diseases, length of hospital stay, duration of hospitalization before positive culture, infection-related laboratory indicators, septic shock, site of infection, hospitalization within the previous 6 months, invasive procedures, immunosuppressive therapy, and antimicrobial exposure.

Laboratory indicators included white blood cell (WBC) count, neutrophil count, hemoglobin level, platelet count, C-reactive protein (CRP), procalcitonin (PCT), and inflammatory cytokines, including interleukin-6 (IL-6), interleukin-8 (IL-8), interleukin-10 (IL-10), interferon-γ (IFN-γ), and tumor necrosis factor-α (TNF-α).

### 2.3. Definitions

The site of infection was determined on the basis of clinical manifestations, imaging findings, laboratory examinations, and microbiological results. Single-system infection was defined as infection mainly confined to one anatomical system or site. Multi-site infection was defined as the simultaneous involvement of two or more anatomical sites or systems during the same episode.

To address the heterogeneity of infection types, infection episodes were further classified into bloodstream infection alone, multi-site infection, and non-bloodstream infection. On the basis of the sites involved, cases were categorized as bloodstream infection alone, intra-abdominal/gastrointestinal tract-related infection, respiratory system-related infection, central nervous system-related infection, and infections involving other sites.

Postoperative or surgery-related status was determined from the documented surgical history, including previous surgery for congenital heart disease, other surgical procedures, and transplantation.

The duration of hospitalization before positive culture was defined as the interval between the date of admission and the date when *E. faecium* or *E. faecalis* was first isolated from a sterile specimen, based on the specimen collection time. A history of hospitalization within 6 months was defined as any hospitalization occurring within 6 months before the current admission.

Invasive procedures mainly included endotracheal intubation, central venous catheterization and drainage tube placement. Endotracheal intubation was analyzed as a binary exposure indicating whether intubation had occurred before the first positive sterile-site culture. The initiation and discontinuation times and cumulative duration of mechanical ventilation before culture positivity were not captured in the study database. Immunosuppressive therapy included chemotherapy, other immunosuppressive treatments, and systemic glucocorticoid exposure. Following the operational definition used by Chusri et al. [16], systemic glucocorticoid exposure was defined as receipt of a prednisolone-equivalent dose of ≥10 mg/day for more than 5 days within the 4 weeks preceding the onset of infection. This criterion was used solely for standardized retrospective exposure classification.

Vancomycin-resistant *enterococci* (VRE) were defined as *E. faecium* or *E. faecalis* classified as resistance to vancomycin according to the guidelines of the Clinical and Laboratory Standards Institute (CLSI) M100 interpretive criteria. Isolates with intermediate susceptibility to vancomycin, if present, were reported separately as vancomycin-non-susceptible *enterococci*.

Because the study period spanned 2018–2025, septic shock was uniformly defined according to the criteria of the International Pediatric Sepsis Consensus Conference (IPSCC) as sepsis accompanied by cardiovascular dysfunction. Cardiovascular dysfunction was defined as persistent hypotension despite adequate fluid resuscitation, a requirement for vasoactive agents to maintain blood pressure, or evidence of inadequate tissue perfusion, including metabolic acidosis, elevated lactate levels, oliguria, prolonged capillary refill time, or an increased core-to-peripheral temperature difference [17].

### 2.4. Pathogen Identification and Antimicrobial Susceptibility Testing (AST)

Sterile specimens were cultured, identified, and tested for antimicrobial susceptibility according to standard procedures in the clinical microbiology laboratory. Matrix-assisted laser desorption/ionization time-of-flight mass spectrometry (MALDI-TOF/MS) (Bruker Daltonics, Bremen, Germany) was used for species-level identification. Antimicrobial susceptibility testing was performed in the microbiology laboratory of the hospital using the Kirby–Bauer disk diffusion method with antimicrobial susceptibility disks (Wenzhou Kangtai Biotechnology Co., Ltd., Wenzhou, China) or the VITEK 2 Compact automated system with AST cards (bioMérieux, Marcy-l’Étoile, France). The antimicrobial agents tested were ampicillin, erythromycin, penicillin, ciprofloxacin, levofloxacin, gentamicin, fosfomycin, minocycline, nitrofurantoin, vancomycin, linezolid, and tigecycline. Antimicrobial susceptibilities were interpreted according to the CLSI M100. Tigecycline susceptibility results for *Enterococcus* species were interpreted according to the European Committee on Antimicrobial Susceptibility Testing Clinical Breakpoint Tables, version 16.0. The reported VRE proportion was calculated only among the included clinical isolates associated with infection. Colonization-screening results, including rectal surveillance cultures, were not included in the study dataset.

### 2.5. Statistical Analysis

Statistical analyses were performed using SPSS Statistics, version 31.0. Continuous variables with a normal distribution were expressed as mean ± standard deviation (mean ± SD), and comparisons between groups were performed using the *t* test. Non-normally distributed continuous variables were expressed as median and interquartile range (IQR) [M (Q1, Q3)], and comparisons between groups were performed using the Mann–Whitney U test. Categorical variables were expressed as numbers and percentages, and comparisons between groups were performed using the χ^2^ test or Fisher’s exact test. Annual trend analysis was performed using the year of specimen collection as the time variable. The χ^2^ test was used to compare differences in species distribution across years, and univariable logistic regression was applied to assess linear trends in the proportion of *E. faecium* and in the distribution of infection types. Considering that both 2018 and 2025 were incomplete calendar years, supplementary analyses were further restricted to the period from 2019 to 2024.

With *E. faecium* coded as the outcome and *E. faecalis* as the reference category, three separate primary binary logistic regression models were fitted to estimate the associations of central venous catheterization, hospitalization for ≥14 days before culture positivity, and endotracheal intubation with pathogen species. Each model was adjusted for septic shock as a clinically important severity covariate. The three exposure variables were analyzed separately in the primary analysis to limit the number of parameters and reduce overadjustment in view of the sample size and their clinical overlap.

Potential collinearity among septic shock, central venous catheterization, hospitalization for ≥14 days before culture positivity, endotracheal intubation, hematologic malignancy, and immunosuppressive therapy was further evaluated using phi correlation coefficients and variance inflation factors (VIFs). In addition, two post hoc sensitivity models were constructed. Both models jointly included septic shock, central venous catheterization, hospitalization for ≥14 days before culture positivity, and endotracheal intubation. Model A additionally included hematologic malignancy, whereas Model B additionally included immunosuppressive therapy. Hematologic malignancy and immunosuppressive therapy were evaluated in separate models because of their strong correlation and elevated VIFs when entered simultaneously. The results of the collinearity diagnostics and sensitivity analyses are provided in Supplementary Tables S1 and S2.

Because length of hospital stay was treated as a count outcome with substantial overdispersion relative to the Poisson distribution, negative binomial regression with a log link was used to estimate the association between pathogen species and length of hospital stay, after adjustment for septic shock. All effect estimates were interpreted as associations rather than causal effects. A two-sided *p* value < 0.05 was considered statistically significant.

## 3. Results

### 3.1. Comparison of Clinical Characteristics Between the E. faecium and E. faecalis Groups

A total of 167 hospitalized children with *Enterococcus* isolated from sterile-site specimens were included in this study. Specimens comprised blood (*n* = 120), tissue (*n* = 19), ascitic fluid (*n* = 15), cerebrospinal fluid (*n* = 5), pleural fluid (*n* = 3), and other drainage fluid (*n* = 5). Among the 167 cases, 98 were infected with *E. faecium* and 69 with *E. faecalis* (Table 1). There was no statistically significant difference in sex distribution between the two groups, with 55 males in the *E. faecium* group and 43 males in the *E. faecalis* group [55/98 (56.12%) vs. 43/69 (62.32%), *p* = 0.423]. The median age was 22.42 (3.41,100.91) months in the *E. faecium* group and 7.35 (2.33, 29.18) months in the *E. faecalis* group, with no statistically significant difference between the groups (*p* = 0.064). The departmental distribution differed significantly between the two groups (*p* < 0.001). In the *E. faecium* group, most patients were from the Hematology-Oncology Department, Pediatric Intensive Care Unit (PICU), and Neonatology Department, comprising 32 cases (32.65%), 28 cases (28.57%), and 19 cases (19.39%), respectively. In contrast, the *E. faecalis* group had the highest proportion of surgical patients, with 28 cases (40.58%), followed by the Neonatology Department with 15 cases (21.74%), PICU with 10 cases (14.49%), and general wards with 10 cases (14.49%).

Regarding underlying diseases, the proportion of children with hematologic malignancies was significantly higher in the *E. faecium* group than in the *E. faecalis* group [46 cases (46.94%) vs. 8 cases (11.59%), *p* < 0.001]. Conversely, the proportion of children who had undergone surgery for congenital heart disease was significantly higher in the *E. faecalis* group than in the *E. faecium* group [20 cases (29.00%) vs. 7 cases (7.14%), *p* < 0.001]. No statistically significant differences were observed between the two groups in the proportions of patients born preterm or patients with other postoperative conditions. The length of hospital stay was longer in the *E. faecium* group than in the *E. faecalis* group [32.00 (23.00,42.00) d vs. 26.50 (21.00,34.00) d, *p* = 0.005]. The duration of hospitalization before culture positivity was 11.00 (6.00,15.00) d in the *E. faecium* group and 10.00 (6.00,12.00) d in the *E. faecalis* group, with no statistically significant difference (*p* = 0.088).

There were no statistically significant differences between the two groups in WBC count, neutrophil count, CRP level, or inflammatory cytokine levels, including IL-6, IL-8, IL-10, IFN-γ, and TNF-α. The PCT level was significantly higher in the *E. faecium* group than in the *E. faecalis* group [1.39 (0.58,2.89) ng/mL vs. 0.49 (0.23,1.25) ng/mL, *p* = 0.001]. The hemoglobin level was lower in the *E. faecium* group than in the *E. faecalis* group [105.00 (86.00,116.00) g/L vs. 108.00 (102.00,116.00) g/L, *p* = 0.026], and platelet count also differed significantly between the groups (*p* = 0.045). There was no statistically significant difference in the proportion of patients with septic shock between the two groups [13 cases (13.27%) vs. 4 cases (5.80%), *p* = 0.116].

Among the 167 pediatric patients with enterococcal infections, 113 cases (67.66%) were classified as bloodstream infection alone, 7 cases (4.19%) as non-bloodstream infection, and 47 cases (28.14%) as multi-site infection (Table 2). The proportion of multi-site infection was higher in the *E. faecium* group than in the *E. faecalis* group [36 cases (36.73%) vs. 11 cases (15.94%), *p* = 0.003]. After classification according to the main involved site, bloodstream infection alone remained the predominant infection type. The cohort included 30 cases with intra-abdominal/gastrointestinal tract-related infection, 13 with respiratory system-related infection, 8 with central nervous system-related infection, and 3 with infections involving other sites. The overall distribution did not differ significantly between the two groups (*p* = 0.201).

Regarding healthcare exposures and invasive procedures, the proportions of hospitalization within the preceding 6 months, central venous catheterization, and immunosuppressive therapy were significantly higher in the *E. faecium* group than in the *E. faecalis* group [59 cases (60.20%) vs. 22 cases (31.88%), *p* < 0.001; 69 cases (70.41%) vs. 24 cases (34.78%), *p* < 0.001; and 46 cases (46.94%) vs. 11 cases (15.94%), *p* < 0.001, respectively]. In contrast, the proportion of endotracheal intubation was higher in the *E. faecalis* group than in the *E. faecium* group [37 cases (53.62%) vs. 34 cases (34.69%), *p* = 0.015]. The frequency of drainage procedures did not differ significantly between the two groups. Regarding antimicrobial exposure before culture positivity, there were no statistically significant differences between the two groups in the use of cephalosporins, carbapenems, β-lactam/β-lactamase inhibitor combinations, vancomycin, penicillins, or linezolid.

### 3.2. Annual Trend

Annual trend analysis showed that the numbers of included cases from 2018 to 2025 were 6, 20, 35, 36, 22, 23, 22, and 3, respectively (Table 3). The proportions of *E. faecium* were 66.67%, 45.00%, 62.86%, 58.33%, 63.64%, 47.83%, 68.18%, and 66.67%, respectively.

Across the full study period, *E. faecium* accounted for 98 of 167 infections (58.68%). There was no statistically significant difference in the distribution of *E. faecium* and *E. faecalis* across different years (χ^2^ = 4.194, *p* = 0.757). Logistic regression using year as a continuous variable did not identify a significant linear trend in the proportion of *E. faecium* (OR = 1.049 per year, 95% CI: 0.879–1.251, *p* = 0.597). When the analysis was restricted to the complete calendar years 2019–2024, the linear trend remained nonsignificant (*p* = 0.504).

Regarding infection types, the proportion of bloodstream infection alone showed an increasing trend from 2018 to 2025 (OR = 1.250 per year, *p* = 0.024), whereas the proportion of multi-site infection showed a decreasing trend (OR = 0.760 per year, *p* = 0.009). However, after the analysis was restricted to the complete calendar years 2019–2024, these trends no longer reached statistical significance (bloodstream infection alone, *p* = 0.065; multi-site infection, *p* = 0.076).

### 3.3. Factors Associated with E. faecium and E. faecalis Infection

Although septic shock was more frequent in the *E. faecium* group than in the *E. faecalis* group [13 cases (13.27%) vs. 4 cases (5.80%)], the between-group difference was not statistically significant (*p* = 0.116). Septic shock was nevertheless retained as a clinically important severity covariate. In three separate primary logistic regression models adjusted for septic shock, central venous catheterization was associated with *E. faecium* infection (aOR = 4.252, 95% CI: 2.172–8.326, *p* < 0.001), as was hospitalization for ≥14 days before culture positivity (aOR = 2.676, 95% CI: 1.202–5.960, *p* = 0.016). Endotracheal intubation was associated with lower odds of *E. faecium* infection (aOR = 0.489, 95% CI: 0.259–0.927, *p* = 0.028), which corresponded to higher odds of *E. faecalis* infection (aOR = 2.043, 95% CI: 1.079–3.867).

The collinearity assessment showed moderate correlations of central venous catheterization with hematologic malignancy (φ = 0.410) and immunosuppressive therapy (φ = 0.439). Hematologic malignancy and immunosuppressive therapy were strongly correlated (φ = 0.906), with VIFs of 5.658 and 6.284, respectively, when all candidate predictors were entered simultaneously. The VIFs of the remaining predictors ranged from 1.132 to 1.593 (Supplementary Table S1).

In sensitivity Model A, which jointly included the healthcare-exposure variables, septic shock, and hematologic malignancy, central venous catheterization remained associated with *E. faecium* infection (aOR = 2.658, 95% CI: 1.238–5.709, *p* = 0.012). The association with hospitalization for ≥14 days before culture positivity was attenuated (aOR = 1.719, 95% CI: 0.708–4.170, *p* = 0.231), as was the inverse association with endotracheal intubation (aOR = 0.852, 95% CI: 0.383–1.898, *p* = 0.695). In sensitivity Model B, which included immunosuppressive therapy instead of hematologic malignancy, central venous catheterization also remained significantly associated with *E. faecium* infection (aOR = 2.995, 95% CI: 1.376–6.516, *p* = 0.006), whereas prolonged pre-culture hospitalization (aOR = 1.710, 95% CI: 0.716–4.084, *p* = 0.227) and endotracheal intubation (aOR = 0.686, 95% CI: 0.300–1.569, *p* = 0.372) were not statistically significant (Supplementary Table S2). These findings indicate that the association with central venous catheterization was comparatively robust, whereas the estimates for prolonged hospitalization and intubation were sensitive to additional adjustment for patient case mix.

In the negative binomial model adjusted for septic shock, *E. faecium* infection was associated with longer hospital stay than *E. faecalis* infection (aIRR = 1.338, 95% CI: 1.160–1.543, *p* < 0.001). All estimates describe associations and do not establish causal effects.

### 3.4. Results of AST

**Figure 1:**
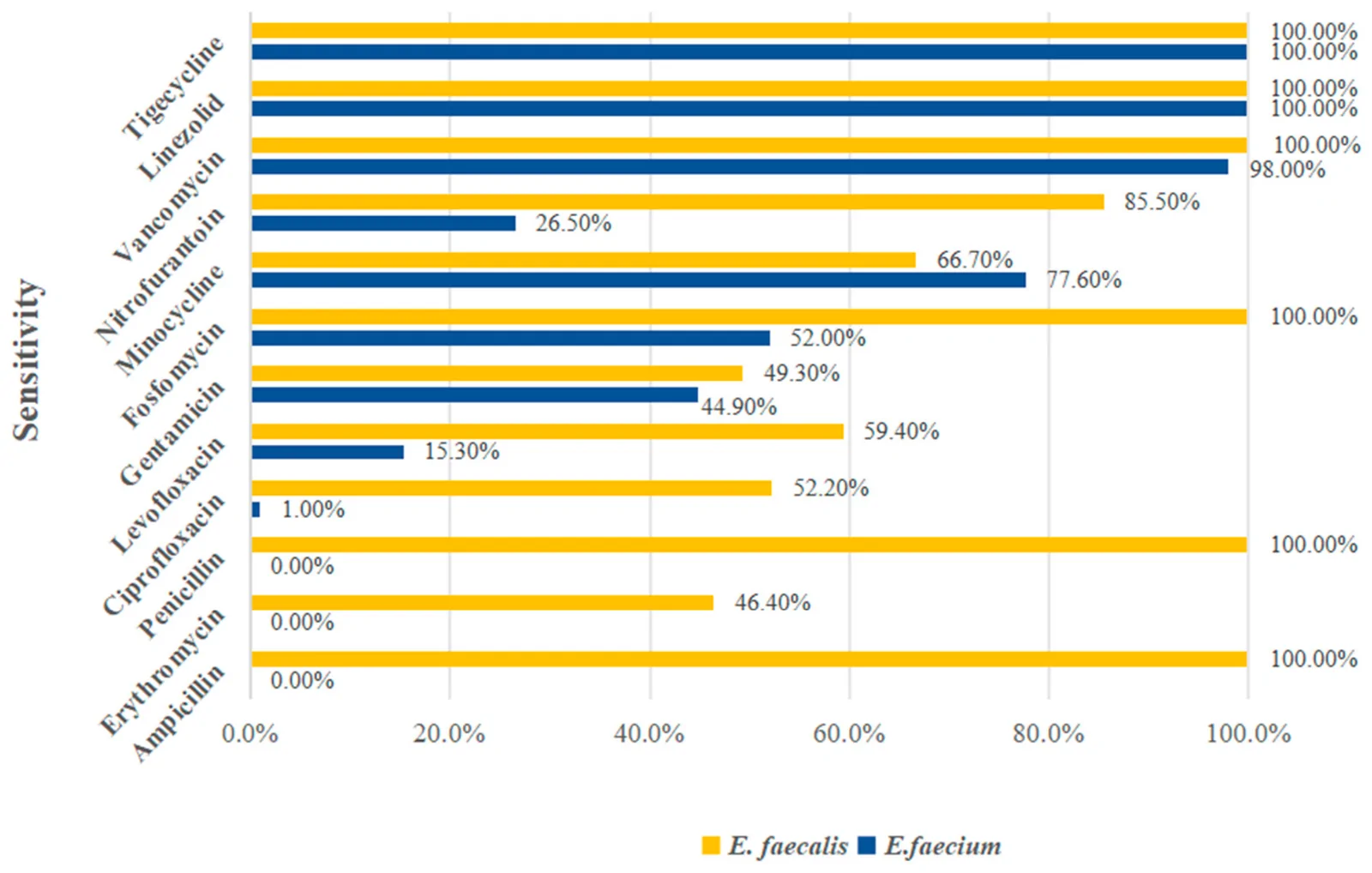
Antimicrobial susceptibility rates among *E. faecium* and *E. faecalis* isolates.

AST showed marked differences between the two enterococcal species (Figure 1). In the *E. faecium* group, the susceptibility rates to ampicillin, erythromycin, and penicillin were 0% for all three agents; the susceptibility rate to ciprofloxacin was 1.0%, and that to levofloxacin was 15.3%. The susceptibility rates to gentamicin, fosfomycin, minocycline, and nitrofurantoin were 44.9%, 52.0%, 77.6%, and 26.5%, respectively. The susceptibility rates to vancomycin, linezolid, and tigecycline were 98.0%, 100%, and 100%, respectively.

In the *E. faecalis* group, the susceptibility rates to ampicillin, penicillin, fosfomycin, vancomycin, linezolid, and tigecycline were 100% for all six agents. The susceptibility rates to erythromycin, ciprofloxacin, levofloxacin, gentamicin, minocycline, and nitrofurantoin were 46.4%, 52.2%, 59.4%, 49.3%, 66.7%, and 85.5%, respectively.

Regarding vancomycin resistance, vancomycin-non-susceptible isolates were uncommon in this cohort. The proportion of VRE isolates was 2.04% in the *E. faecium* group and 0% in the *E. faecalis* group, with an overall VRE proportion of 1.20% among all enterococcal isolates.

## 4. Discussion

This single-center retrospective study identified differences between pediatric *E. faecium* and *E. faecalis* infections in host characteristics, healthcare exposures, invasive procedures, antimicrobial susceptibility, and clinical outcomes. *E. faecium* infection was observed more frequently among children with hematologic malignancies, immunosuppression, PICU admission, prior hospitalization, and central venous catheterization, and was associated with higher PCT levels, a higher proportion of multi-site infections, and longer hospitalization. *E. faecalis* infection was observed more frequently in surgical patients and children who had undergone surgery for congenital heart disease, and was associated with endotracheal intubation. These patterns may reflect differences in patient case mix and healthcare exposure; given the observational design, they should not be interpreted as evidence that any individual exposure caused infection by a particular species.

Recent studies have suggested that the clinical importance of *E. faecium* is related to its capacity to acquire antimicrobial resistance genes, adapt to the healthcare environment, and tolerate disinfectant and antimicrobial pressures [1,2,4,13]. In the present study, the *E. faecium* group had a higher prevalence of hematologic malignancy, immunosuppressive therapy, hospitalization within the preceding 6 months, central venous catheterization, and hospitalization for ≥14 days before culture positivity. Children with hematologic malignancies often experience chemotherapy-associated mucosal barrier injury, neutropenia, repeated hospitalizations, long-term central venous catheterization, and broad-spectrum antimicrobial exposure, which may contribute to intestinal dysbiosis, enterococcal colonization, mucosal translocation, or catheter-related bloodstream infection [7,8,9,13,18]. The association between central venous catheterization and *E. faecium* infection remained statistically significant in both sensitivity models incorporating either hematologic malignancy or immunosuppressive therapy. Nevertheless, the attenuation of the aOR from 4.252 in the primary severity-adjusted model to 2.658–2.995 in the sensitivity models suggests that part of the observed association was explained by the underlying disease and treatment context. Central venous catheterization should therefore be interpreted as a composite marker of vascular access, treatment intensity, and prolonged healthcare exposure, and potentially as a mediator on the clinical pathway from severe underlying disease to invasive infection, rather than as an isolated causal risk factor. In high-risk children with *E. faecium* isolated from blood or another sterile site, the findings support careful assessment of catheter necessity, possible catheter-related infection, persistent bacteremia, multi-site involvement, and relevant infection-control measures.

No significant between-group differences were observed in binary measures of exposure to the recorded antimicrobial classes before culture positivity, particularly for carbapenems, β-lactam/β-lactamase inhibitor combinations, vancomycin, and linezolid. However, this does not indicate that antimicrobial exposure is unimportant in the development of *E. faecium* infection. In this retrospective study, antimicrobial exposure was analyzed only as a binary indicator of whether a given antimicrobial class had been used, without further quantification of days of therapy (DOT), cumulative dose, intensity of combination therapy, spectrum of antimicrobial coverage, antimicrobial exposure during previous hospitalizations, or frequency of antimicrobial switching. The *E. faecium* group more often had a history of hospitalization within the preceding six months, hospitalization for ≥14 days before culture positivity, immunosuppression, and central venous access exposure, all of which collectively indicate more prolonged and complex exposure to the healthcare environment. Previous studies have shown that prior exposure to broad-spectrum antimicrobial agents, such as piperacillin/tazobactam and carbapenems, is associated with an increased risk of *E. faecium* bacteremia, suggesting that antimicrobial selective pressure may promote ecological replacement of *E. faecium* relative to *E. faecalis* and facilitate its clinical emergence [19]. Therefore, the effect of antimicrobial exposure may not be captured by differences in a single antimicrobial class, but rather may interact with prolonged hospitalization, underlying diseases, invasive procedures, and nosocomial microbiota pressure to shape the clinical context for *E. faecium* infection [7,19]. Future studies should further incorporate DOT, indices of combined antimicrobial exposure, and dynamic monitoring of intestinal colonization to more accurately evaluate the relationship between antimicrobial selective pressure and infections caused by different Enterococcus species.

The antimicrobial susceptibility results underscore the potential clinical relevance of species identification. At our center, *E. faecium* susceptibility rates to ampicillin and penicillin were 0%, whereas *E. faecalis* susceptibility rates to both agents were 100%. Recent surveillance data have also shown that *E. faecium* generally exhibits a high rate of ampicillin resistance. According to the 2025 CHINET China Antimicrobial Resistance Surveillance Network data, the ampicillin resistance rate among *E. faecium* isolates was 92.4%, markedly higher than the rates among *E. faecalis* isolates at 1.5% (www.chinets.com, accessed on 30 April 2026). A global systematic review and meta-analysis published in 2025 reported pooled resistance rates of *E. faecium* to ampicillin and penicillin of 58.9% and 71.9%, respectively, with the ampicillin resistance rate increasing to 72.3% during 2020–2022 [20]. In addition, studies of ICU patients in China have reported resistance rates of *E. faecium* to penicillin and ampicillin as high as 100% [21]. Therefore, the absence of *E. faecium* isolates susceptible to ampicillin or penicillin in this study (0% susceptibility) represents an extreme local non-susceptibility profile compared with national and global surveillance estimates, but is consistent with the high level of resistance reported in critically ill patients, patients with prolonged hospitalization, and populations with substantial healthcare exposure. These findings suggest that this resistance profile among pediatric *E. faecium* isolates at our center may reflect intense β-lactam selective pressure. At the molecular level, ampicillin and penicillin resistance in *E. faecium* is primarily associated with the low affinity of PBP5 for β-lactams; high-level resistance has been linked to resistance-associated pbp5 alleles and amino-acid substitutions in its C-terminal transpeptidase domain, as well as increased pbp5/PBP5 expression [4,22,23]. However, because pbp5 sequencing, expression analysis, and whole-genome typing were not performed, these mechanisms remain biologically plausible explanations rather than demonstrated mechanisms in our isolates, and the possible dissemination of specific resistant clones cannot be determined. Future studies integrating these approaches are needed to clarify both the resistance mechanism and local transmission dynamics. Clinically, if *E. faecium* is isolated from sterile sites in high-risk children, clinicians should be alert to the risk of inadequate empirical coverage with ampicillin or penicillin. In contrast, for *E. faecalis* infections with confirmed susceptibility, narrow-spectrum β-lactam agents may still be preferentially considered for targeted therapy [12,22,24]. Therefore, species identification not only informs etiological classification but also has direct implications for the breadth of empirical antimicrobial coverage, de-escalation strategies for targeted therapy, and antimicrobial stewardship. However, the findings from this single-center study should not be taken as a general recommendation for empirical therapy. Once isolate-specific susceptibility is available, treatment can be narrowed or otherwise adjusted in accordance with established guidance and antimicrobial-stewardship principles.

We reviewed current international guidance relevant to the treatment of resistant enterococcal infections. For enterococcal catheter-related bloodstream infection, the IDSA guideline recommends ampicillin for ampicillin-susceptible isolates, vancomycin for ampicillin-resistant isolates, and linezolid or daptomycin for isolates resistant to both ampicillin and vancomycin, based on isolate-level susceptibility [15]. The 2023 European Society of Cardiology guideline provides infection-site-specific recommendations for enterococcal infective endocarditis and emphasizes expert-directed management of multidrug-resistant isolates [25]. Accordingly, treatment of pediatric invasive enterococcal infection should be individualized according to species, isolate-level susceptibility, infection site, source control, patient age, organ function, and antimicrobial pharmacokinetics and safety, preferably with pediatric infectious-diseases consultation. Because the present study was not designed to compare treatment regimens or clinical efficacy, our susceptibility findings should not be interpreted as supporting a universal empirical regimen.

The marked between-species differences in antimicrobial susceptibility observed in this study underscore the clinical value of rapid discrimination between *E. faecalis* and *E. faecium*. MALDI-TOF/MS can rapidly identify cultured isolates, and validated direct-from-positive-blood-culture workflows may further shorten turnaround time [26]. Multiplex polymerase chain reaction (PCR) blood-culture panels can identify both species and detect selected resistance determinants, particularly vanA and vanB, supporting earlier optimization or de-escalation of empirical therapy [27]. Emerging species-specific real-time PCR and isothermal amplification platforms may allow for more rapid, less equipment-dependent detection; a recent multiplex recombinase polymerase amplification assay with lateral-flow readout detected both species together with vanA and vanB, although broader clinical validation remains necessary [28]. In contrast, enzyme-linked immunosorbent assay (ELISA)-based assays are not currently validated or standardized for routine differentiation of *E. faecalis* from *E. faecium* and remain investigational. Rapid assays are target- and platform-dependent and do not replace culture-based susceptibility testing. Their greatest value is likely to arise when species and resistance-marker results are interpreted with phenotypic susceptibility data within antimicrobial-stewardship pathways.

Children in the *E. faecium* group had greater healthcare exposure, a more resistant antimicrobial phenotype, higher PCT levels, a higher proportion of multi-site infection, and longer hospitalization. The adjusted negative binomial model also identified an association between *E. faecium* infection and length of stay. Because species, underlying disease, invasive procedures, prior therapy, and duration of hospitalization are temporally and clinically interrelated, this association should not be interpreted as evidence that *E. faecium* itself caused the additional hospital days. Reverse causation and residual confounding remain plausible.

In the primary severity-adjusted analysis, endotracheal intubation was associated with *E. faecalis* infection. However, this association was attenuated and was no longer statistically significant after additional adjustment for hematologic malignancy or immunosuppressive therapy. This finding suggests that the original association was partly explained by differences in patient case mix and should be considered exploratory. Endotracheal intubation was recorded only as a binary exposure, and the timing and cumulative duration of mechanical ventilation before culture positivity were unavailable. Therefore, the observed association cannot be interpreted as an independent causal effect of intubation. The present study included monomicrobial infections identified from sterile-site specimens and did not include respiratory-tract cultures. Thus, this finding neither indicates a respiratory origin of infection nor distinguishes among intestinal translocation, surgical-site infection, catheter-related dissemination, or other potential routes. Endotracheal intubation is therefore best regarded as a nonspecific marker of perioperative critical-care intensity, artificial-airway exposure, and overall treatment complexity [29]. The *E. faecalis* group included a higher proportion of surgical patients and children who had undergone surgery for congenital heart disease. These patients may experience anesthesia, cardiopulmonary bypass, hemodynamic fluctuations, tissue hypoperfusion, mechanical ventilation, postoperative intensive care, altered nutritional support, and perioperative antimicrobial exposure, which could theoretically disrupt the intestinal microbiota and impair mucosal barrier integrity [5,30,31]. Recent pediatric studies provide further, although inconsistent, support for this biological framework. In the prospective GuMiBear cohort, infants with congenital heart disease had an altered gut microbiome compared with healthy controls and showed postoperative enrichment of *Enterococcus* and several other potentially pathogenic genera; however, only 13 paired samples were available and overall pre- to postoperative differences did not reach statistical significance [32]. A 2025 study of children with ventricular septal defect or tetralogy of Fallot similarly identified preoperative enrichment of *Enterobacteriaceae* and Shigella with depletion of Bifidobacterium, but found no significant early community-level change after cardiopulmonary bypass and observed recovery toward a control-like microbiota with increased short-chain fatty acid-producing taxa approximately 1 year after surgery [33]. Together, these data indicate that pediatric congenital heart disease is associated with perioperative gut dysbiosis and that microbiome composition may evolve after extracorporeal circulation, while also underscoring the heterogeneity and small sample sizes of the available studies. Nevertheless, the proposed “surgical stress–intestinal barrier injury–microbial translocation” pathway remains a biologically plausible hypothesis rather than a mechanism demonstrated by the present data. When *E. faecalis* is isolated from a child after surgery, clinical assessment should therefore integrate the type and complexity of surgery, cardiopulmonary-bypass and perfusion status, gastrointestinal function, nutritional support, invasive devices, and the documented site of infection [34].

The low VRE frequency in this cohort warrants cautious interpretation. Only two of 98 *E. faecium* isolates (2.04%) and none of 69 *E. faecalis* isolates were vancomycin-resistant, despite the high prevalence of hematologic malignancy and frequent previous exposure to broad-spectrum agents. Compared with reports from other tertiary and high-risk pediatric populations [7,9,20], this finding may reflect low local VRE endemicity or limited circulation of resistant clones and could also be influenced by the surrounding infection-prevention and antimicrobial-stewardship environment. However, the study did not collect program-level data on the content, intensity, adherence, or temporal implementation of internal infection-control policies, nor did it include molecular surveillance. Consequently, it remains unknown whether the two VRE isolates were sporadic or clonally related and whether hospital-associated *E. faecium* lineages, including those historically grouped within clonal complex 17 (CC17), were circulating at the center. Accordingly, the low VRE rate cannot be attributed causally to institutional policies or interpreted as evidence of their effectiveness. Nevertheless, the findings support incorporating guideline-based measures into local protocols. Standard Precautions, particularly hand hygiene, should be applied to all patients. For children colonized or infected with vancomycin-resistant enterococci or other epidemiologically important multidrug-resistant enterococci, Contact Precautions and single-room placement should be implemented according to institutional risk assessment and local policy; when a single room is unavailable, cohorting may be considered in consultation with the infection-prevention team. These measures should be supported by gloves and gowns for room entry, dedicated or appropriately disinfected equipment, and enhanced environmental cleaning [35,36,37]. During suspected clusters or outbreaks, active surveillance and molecular epidemiologic investigation may help characterize transmission and target interventions. Antimicrobial stewardship should include leadership accountability, prospective audit and feedback and/or formulary preauthorization, use of the local antibiogram, timely species identification and susceptibility testing, prompt de-escalation to the narrowest active agent, and optimization of dose and duration [38]. Because isolation strategies vary with the local prevalence of vancomycin-resistant enterococci, resources, and transmission risk, these measures should be operationalized through institutional infection-prevention and antimicrobial-stewardship teams rather than extrapolated as a universal protocol from this single-center study.

This study has several limitations. First, the sample was relatively small and was drawn from a single tertiary pediatric center. Selection bias, departmental case mix, referral patterns, local prescribing practices, and infection-prevention policies may limit precision and external validity; the findings require validation in larger multicenter cohorts. Importantly, the cohort included clinically heterogeneous populations, such as preterm infants, children with hematologic malignancies, and children with congenital heart disease or other surgical conditions. Age, developmental stage, underlying disease, repeated hospitalization, invasive procedures, and treatment intensity were closely interrelated and may have introduced confounding that could not be fully addressed by the available sample size. Although age did not differ significantly between the two species groups in the overall comparison, the study was not powered for age-stratified or disease-specific analyses; direct comparison across these clinically distinct subgroups should therefore be interpreted cautiously. Future multicenter studies should prespecify age categories and underlying-condition strata and evaluate whether associations differ among major pediatric subgroups. Second, several exposures were clinically interrelated, including prolonged hospitalization, central venous catheterization, immunosuppressive therapy, and hematologic malignancy. Collinearity diagnostics demonstrated a particularly strong correlation between hematologic malignancy and immunosuppressive therapy. Although these variables were therefore examined in separate sensitivity models, residual confounding and mediation could not be fully distinguished. The association between central venous catheterization and *E. faecium* infection remained statistically significant in both sensitivity models, but the associations involving prolonged hospitalization and endotracheal intubation were attenuated and no longer statistically significant. These latter findings should consequently be interpreted as exploratory. Endotracheal intubation was recorded only as a binary exposure; its timing and the cumulative duration of mechanical ventilation before culture positivity were not captured and therefore could not be adjusted. Detail of surgical procedures, surgical complexity, cardiopulmonary-bypass duration, and perioperative hemodynamic variables were also unavailable. Residual confounding by surgical case mix and perioperative critical-care intensity therefore remains possible. Prospective studies with larger samples, time-dependent exposure measurements, and a prespecified causal framework are required to distinguish direct effects from proxy or mediating relationships. Third, previous antimicrobial exposure was captured only as a binary indicator for each drug class. The absence of duration, cumulative dose, timing, combination intensity, spectrum-weighted exposure, and prior-admission exposure precluded a comprehensive assessment of antimicrobial pressure and may have caused nondifferential misclassification. Fourth, patients with polymicrobial infections were excluded, and respiratory-tract specimens were not included. Although these criteria improved analytic specificity for monomicrobial infections identified from sterile sites, they reduced applicability to routine postoperative and intra-abdominal infections and prevented determination of a respiratory origin or other route of infection. In addition, the VRE estimate was based on clinically adjudicated infection isolates from sterile sites rather than systematic hospital-wide or rectal colonization surveillance. Colonization isolates, non-sterile-site isolates, polymicrobial episodes, and repeat isolates beyond the first qualifying isolate were not represented. Consequently, the 2.04% VRE proportion describes this selected invasive-infection cohort and may underestimate the hospital’s overall VRE colonization or infection burden. Fifth, no molecular typing or sequencing was performed. The absence of multilocus sequence typing (MLST), pulsed-field gel electrophoresis (PFGE), or whole-genome sequencing (WGS) precluded assessment of whether hospital-associated E. faecium lineages, including those historically grouped within clonal complex 17 (CC17), were present; whether the two VRE isolates were clonally related; and whether healthcare transmission had occurred. This is a critical limitation. Future prospective studies should therefore treat molecular epidemiology as an essential, prespecified component rather than an optional ancillary analysis. Systematic colonization surveillance should be combined with isolate preservation and WGS-based phylogenetic analysis, supplemented by MLST and/or PFGE where appropriate, to distinguish sporadic infections from clonal transmission and to link resistance and virulence determinants to specific lineages. These molecular data should be integrated with detailed antimicrobial-exposure metrics and infection-control information.

## 5. Conclusions

In this single-center cohort, pediatric *E. faecium* and *E. faecalis* infections were associated with distinct patient profiles, healthcare exposures, susceptibility patterns, and measures of disease burden. No significant linear change in species distribution was identified over the study period. *E. faecium* was more often observed among children with immunocompromising conditions, prolonged hospitalization, and central venous access, whereas *E. faecalis* was more frequent in surgical settings and among children who had undergone surgery for congenital heart disease. Species identification may complement patient-level assessment and the current local antibiogram while isolate-specific susceptibility results are pending; it should not be used alone to determine empirical therapy. These observational associations do not establish causality and should be generalized with caution until confirmed in larger multicenter studies that include polymicrobial infections and more detailed antimicrobial exposure data. Prospective multicenter studies should also assess whether rapid species-identification workflows integrated with antimicrobial stewardship shorten the time to optimized therapy and improve patient-centered outcomes.

## Figures and Tables

**Table 1:** Comparison of the clinical characteristics between the *E. faecium* and *E. faecalis* groups.

Variables	*E. faecium* (*n* = 98)	*E. faecalis* (*n* = 69)	Test Statistic	*p* Value
Male	55 (56.12%)	43 (62.32%)	0.641	0.423
Age, m, median (IQR)	22.42 (3.41, 100.91)	7.35 (2.33, 29.18)	1.853	0.064
Department			28.957	<0.001
Neonatology Department	19 (19.39%)	15 (21.74%)		
Hematology-Oncology Department	32 (32.65%)	6 (8.70%)		
PICU	28 (28.57%)	10 (14.49%)		
Surgery Department	14 (14.29%)	28 (40.58%)		
General wards	5 (5.10%)	10 (14.49%)		
Underlying diseases				
Hematologic malignancy	46 (46.94%)	8 (11.59%)	23.119	<0.001
Preterm birth	14 (14.29%)	9 (13.04%)	0.053	0.819
Previous surgery for congenital heart disease	7 (7.14%)	20 (29.00%)	14.253	<0.001
Other postoperative status	20 (20.41%)	13 (18.84%)	0.063	0.846
Length of hospital stay, d, median (IQR)	32.00 (23.00, 42.00)	26.50 (21.00, 34.00)	2.834	0.005
Duration of hospitalization before culture positivity, d, median (IQR)	11.00 (6.00, 15.00)	10.00 (6.00, 12.00)	1.707	0.088
WBC, ×10^9^/L, median (IQR)	12.36 (3.20, 17.09)	12.34 (9.36, 12.35)	1.341	0.180
Neutrophil count, ×10^9^/L, mean ± SD	7.65 ± 5.74	8.55 ± 4.18	1.166	0.245
Hemoglobin, g/L, median (IQR)	105.00 (86.00, 116.00)	108.00 (102.00, 116.00)	2.231	0.026
Platelet count, ×10^9^/L, median (IQR)	323.00 (62.75, 402.75)	319.00 (267.75, 364.25)	2.009	0.045
CRP, mg/L, median (IQR)	74.00 (45.00, 1025.25)	58.50 (46.00, 96.50)	0.579	0.563
PCT, ng/mL, median (IQR)	1.39 (0.58, 2.89)	0.49 (0.23, 1.25)	3.316	0.001
IL-6, pg/mL, median (IQR)	502.15	649.07	0.832	0.406
	(69.83, 2554.89)	(237.82, 2849.02)		
IL-8, pg/mL, median (IQR)	248.58 (32.16, 858.53)	242.66 (77.45, 960.76)	0.663	0.507
IL-10, pg/mL, median (IQR)	77.47 (21.84, 225.92)	86.65 (42.72, 175.05)	0.663	0.507
IFN-γ, pg/mL, median (IQR)	43.88 (17.91, 76.68)	37.80 (13.95, 60.32)	0.942	0.346
TNF-α, pg/mL, median (IQR)	83.61 (32.72, 143.48)	94.22 (50.41, 158.49)	1.475	0.140
Septic shock	13 (13.27%)	4 (5.80%)	2.470	0.116
Infection site			8.656	0.003
Single-system infection	62 (63.27%) 36 (36.73%)	58 (84.06%) 11 (15.94%)		
Multi-site infection				
Hospitalization within the preceding 6 months	59 (60.20%)	22 (31.88%)	13.002	<0.001
Endotracheal intubation	34 (34.69%)	37 (53.62%)	5.937	0.015
Central venous catheterization	69 (70.41%)	24 (34.78%)	20.826	<0.001
Drainage procedure	20 (20.41%)	9 (13.04%)	1.530	0.216
Immunosuppressive therapy	46 (46.94%)	11 (15.94%)	17.304	<0.001
Cephalosporins	65 (66.33%)	37 (53.62%)	2.749	0.097
Carbapenems	55 (56.12%)	39 (56.52%)	0.003	0.959
β-lactam/β-lactamase inhibitor combinations	52 (53.06%)	43 (62.32%)	1.415	0.234
Vancomycin	19 (19.38%)	16 (23.19%)	0.353	0.552
Penicillins	14 (14.29%)	15 (21.74%)	1.568	0.211
Linezolid	13 (13.27%)	7 (10.14%)	0.374	0.541

Abbreviations: IQR, interquartile range; PICU, Pediatric intensive care unit; WBC, white blood cell; CRP, C-reactive protein; PCT, procalcitonin; IL-6, interleukin-6; IL-8, interleukin-8; IL-10, interleukin-10; IFN-γ, interferon-γ; TNF-α, Tumor necrosis factor-α.

**Table 2:** Distribution of *E. faecium* and *E. faecalis* according to major infection type.

Infection Type	*E. faecium* (*n* = 98)	*E. faecalis* (*n* = 69)	Total
Bloodstream infection alone	62 (63.27%)	51 (73.91%)	113
Intra-abdominal/gastrointestinal tract-related infection	18 (18.37%)	12 (17.39%)	30
Respiratory system-related infection	11 (11.22%)	2 (2.90%)	13
Central nervous system-related infection	6 (6.12%)	2 (2.90%)	8
Infection involving other sites	1 (1.02%)	2 (2.90%)	3

**Table 3:** Annual changes in species distribution and infection types from 2018 to 2025.

Year	Total Cases	*E. faecium*	*E. faecalis*	Bloodstream Infection Alone	Multi-Site Infection	Non-Bloodstream Infection
2018	6	4 (66.67%)	2 (33.33%)	2 (33.33%)	4 (66.67%)	0 (0.00%)
2019	20	9 (45.00%)	11 (55.00%)	12 (60.00%)	8 (40.00%)	0 (0.00%)
2020	35	22 (62.86%)	13 (37.14%)	20 (57.14%)	14 (40.00%)	1 (2.86%)
2021	36	21 (58.33%)	15 (41.67%)	25 (69.44%)	8 (22.22%)	3 (8.33%)
2022	22	14 (63.64%)	8 (36.36%)	18 (81.82%)	2 (9.09%)	2 (9.09%)
2023	23	11 (47.83%)	12 (52.17%)	18 (78.26%)	5 (21.74%)	0 (0.00%)
2024	22	15 (68.18%)	7 (31.82%)	16 (72.73%)	6 (27.27%)	0 (0.00%)
2025	3	2 (66.67%)	1 (33.33%)	2 (66.67%)	0 (0.00%)	1 (33.33%)

## Data Availability

The original contributions presented in the study are included in the article. Further inquiries can be directed to the corresponding author.

## Supplementary Materials

The following supporting information can be downloaded at https://www.mdpi.com/article/10.3390/pathogens15090927/s1, Table S1: Phi correlation coefficients and VIFs for candidate predictors; Table S2: Comorbidity-adjusted sensitivity analyses for factors associated with *E. faecium* infection.

## Abbreviations

The following abbreviations are used in this manuscript:

AST	Antimicrobial Susceptibility Testing
CRP	C-reactive protein
DOT	Days of therapy
ELISA	Enzyme-linked immunosorbent assay
IFN-γ	Interferon-γ
IL-6	Interleukin-6
IL-8	Interleukin-8
IL-10	Interleukin-10
IQR	Interquartile range
PCR	Polymerase Chain Reaction
PCT	Procalcitonin
PICU	Pediatric intensive care unit
TNF-α	Tumor necrosis factor-α
VIFs	Variance inflation factors
VRE	Vancomycin-resistant *Enterococcus*
WBC	White blood cell
