# Supplementary material for: Clinical Characteristics and Antimicrobial Resistance Profiles of Pediatric Enterococcal Infections: A Comparison Between Enterococcus faecium and Enterococcus faecalis
Source: Pathogens. 2026 Sep 3;15(9):927. doi: 10.3390/pathogens15090927 (PMC13610483; doi:10.3390/pathogens15090927)
Supplement: Supplementary file 1 [file pathogens-15-00927-s001.zip › pathogens-4505421-supplementary.pdf]

Supplementary Table S1

Phi correlation coefficients and VIFs for candidate predictors

| Variable | Septic shock | Central venous catheterization | Hospitalization ≥14d before culture positivity | Endotracheal intubation | Hematologic malignancy | Immunosuppressive therapy | VIF |
|---|---|---|---|---|---|---|---|
| Septic shock | 1.000 | | | | | | 1.151 |
| Central venous catheterization | 0.221 | 1.000 | | | | | 1.433 |
| Hospitalization ≥14d before culture positivity | 0.170 | 0.323 | 1.000 | | | | 1.132 |
| Endotracheal intubation | −0.169 | −0.062 | −0.080 | 1.000 | | | 1.593 |
| Hematologic malignancy | 0.318 | 0.410 | 0.189 | −0.543 | 1.000 | | 5.658 |
| Immunosuppressive therapy | 0.342 | 0.439 | 0.194 | −0.568 | 0.906 | 1.000 | 6.284 |

Note: All variables were binary; therefore, Pearson correlations are equivalent to phi coefficients. VIFs were calculated with all six candidate predictors entered simultaneously.

Supplementary Table S2

Comorbidity-adjusted sensitivity analyses for factors associated with *E. faecium* infection

| Variable | Model A, aOR (95% CI) | P value | Model B, aOR (95% CI) | *P* value |
|---|---|---|---|---|
| Septic shock | 0.775 (0.199–3.021) | 0.714 | 0.905 (0.240–3.408) | 0.883 |
| Central venous catheterization | 2.658 (1.238–5.709) | 0.012 | 2.995 (1.376–6.516) | 0.006 |
| Hospitalization ≥14 d before culture positivity | 1.719 (0.708–4.170) | 0.231 | 1.710 (0.716–4.084) | 0.227 |
| Endotracheal intubation | 0.852 (0.383–1.898) | 0.695 | 0.686 (0.300–1.569) | 0.372 |
| Hematologic malignancy | 4.182 (1.455–12.017) | 0.008 | — | — |
| Immunosuppressive therapy | — | — | 2.250 (0.801–6.321) | 0.124 |

Note: The dependent variable was *E. faecium* infection, with *E. faecalis* as the reference category. Model A included hematologic malignancy; Model B included immunosuppressive therapy. The maximum VIFs were 1.853 and 2.058 in Models A and B, respectively.
